# Outcomes of a computer-based cognitive training (CoRe) in early phases of cognitive decline: a data-driven cluster analysis

**DOI:** 10.1038/s41598-022-26924-2

**Published:** 2023-02-07

**Authors:** Sara Bernini, Alessia Gerbasi, Silvia Panzarasa, Silvana Quaglini, Matteo Cotta Ramusino, Alfredo Costa, Micol Avenali, Cristina Tassorelli, Tomaso Vecchi, Sara Bottiroli

**Affiliations:** 1 IRCCS Mondino Foundation, 27100 Pavia, Italy; 2 Department of Electrical, Computer and Biomedical Engineering, University of Pavia, 27100 Pavia, Italy; 3 Department of Brain and Behavioral Sciences, University of Pavia, 27100 Pavia, Italy; 4 Faculty of Law, Giustino Fortunato University, 82100 Benevento, Italy

**Keywords:** Psychology, Human behaviour

## Abstract

The present study aimed to identify clusters of cognitive profiles as well as to explore the effects of these clusters on demographic/individual characteristics and on improvements after a computer-based cognitive training (CCT) in early cognitive impairment. Fifty-seven subjects underwent to an adaptive CCT for 3 weeks (4 individual face-to-face sessions/week of 45 min) and were evaluated at baseline (T0), post-intervention (T1), and after 6 (T2) and 12 (T3) months. Clusters of cognitive profiles were explored with k-means analysis. The analysis revealed two clusters, which were composed by 27 and 30 patients characterized by lower (Cluster 1) and higher (Cluster 2) cognitive functioning. At T1, cognitive performance improved in both groups, but Cluster 1 gained more benefits in global cognitive functioning than Cluster 2. However, at T3, Cluster 2 remained stable in its clinical condition, whereas Cluster 1 showed a pronounced worsening. In conclusion, Cluster 1 profile was associated with a more marked but also short-lasting responsiveness to CCT, whereas patients fitting with Cluster 2 characteristics seemed to obtain more CCT benefits in terms of stability or even delay of cognitive/functional decline. These findings may have relevant implications in informing the timing and modality of delivery of CCT.

## Introduction

Previous studies demonstrated the effectiveness of Cognitive Training (CT) programs in patients in the early stage of cognitive decline, i.e., mild cognitive impairment (MCI) and mild dementia^1,2^. Traditionally, CT consists of in-person sessions usually administered in hospital setting under therapist monitoring by using paper-and-pencil techniques and, more recently, technology-based solutions (e.g., computer-based CT–CCT). However, not all the patients involved in this kind of interventions obtain the same benefits, probably because of the great inter-individual variability in the elderly^3^. Literature in this field suggests indeed that some demographic/individual characteristics (such as age, education, cognitive reserve (CR), and baseline cognitive profiles) are likely associated with better interventions outcomes. Thus, it may be helpful to consider these features for stratifying patients according to their chance of benefiting from CT^4–6^ or CCT^7,8^. To shed light on this argument, even if developed in the field of CT, two opposite models have been proposed^9^. According to the *compensation* model, these activities appear to allow participants with low performances to “compensate” for their weaknesses. That is, overall individual differences among participants at baseline are reduced after intervention. By contrast, according to the *magnification* model, individual differences are “magnified” after CT. This means that individuals with the highest levels of baseline performance are those reaching benefits that are more substantial. However, results are still heterogeneous^10–13^ (and only in few cases considering CCT^14,15^ or pathological aging^16–18^), highlighting the need to further explore patients’ individual characteristics and their role as putative predictors of response to CCT in the field of neurodegenerative diseases.

The present study had a twofold aim. First, to identify and describe subgroups of early-impaired patients with higher vs lower cognitive profiles using an unsupervised clustering technique. Second, to explore how the identified clusters differ in terms of: (a) demographic and individual characteristics, (b) performance at the CCT tasks, (c) immediate (T1) and maintenance at 6 (T2) and 12 (T3) months of global cognitive functioning improvement following CCT, and (d) disease progression after 1 year. For clustering, we selected two specific cognitive abilities, that is, global cognitive functioning and processing speed (PS). Global cognitive functioning was evaluated with the Mini Mental State Examination (MMSE), which is the most used screening test in the field of neurodegenerative diseases, and represents an index of global status comprising all cognitive abilities^19^. PS is a lower order cognitive function that is necessary for higher-order cognitive domains^20^ and it is often considered as a principal marker of decline in fluid abilities^21,22^. Thus, PS may be the source of deficits in other areas of cognition. The identification of PS deficits is particularly important since this cognitive ability can be sensitive to the effects of CCT^23,24^, given that here exercises to be performed are typically timed^7,25^.

In the context of the present study, as CCT intervention, we used CoRe (Cognitive Rehabilitation)^26,27^, a software for face-to-face individual sessions developed by clinicians and bioengineers and successfully tested in patients with different types and levels of cognitive impairment due to neurodegenerative diseases^28–30^. Here, we showed that CoRe resulted significantly effective in improving cognitive functioning also when compared to a traditional paper-and-pencil CT, this thanks to its adaptive approach (i.e., task difficulty was adjusted depending on the subject’s performance)^31^. Moreover, given that CoRe is an ontology-based software tool^32^, it allows several degrees of personalization and the possibility to generate different patient-tailored exercises.

In the present study, we expected that patients having higher cognitive profiles would present protective demographic and individual baseline characteristics, including high levels of CR. This is because CR is a theoretical construct that derives from the contribute of several factors (e.g., education, occupational attainment and leisure time activities^33^) that may be involved in explaining the discrepancy between clinical manifestations and the underlying cerebral pathology^34^. By contrast, we expected that patients with lower cognitive profiles would be characterized by an opposite set of individual characteristics. Finally, we also hypothesized that higher and lower cognitive profiles may have a different trend of post-treatment improvement.

## Results

### Cluster analysis

The best silhouette score (0.55) was obtained with k = 2 clusters. Twenty-seven and thirty patients composed the resulting clusters. Centroids for Cluster 1 were MMSE = − 0.961 ± 0.502, PS = 0.135 ± 0.452; whereas for Cluster 2 were MMSE = 0.763 ± 0.433, PS = − 0.182 ± 0.450. The values of the centroids show that Cluster 1 had a worse cognitive profile than Cluster 2.

### Demographic and individual characteristics of the two clusters

Table 1 reports the results of the statistical tests comparing the two clusters at the baseline (T0). As evident, there was no significant difference in terms of age (U = 375.50, *p* = 0.321), while Cluster 2 reported a significantly higher level of education (U = 240.00, 0.006) than Cluster 1. In addition, the two clusters were significantly different in terms of gender (χ^2^ = 6.23, *p* = 0.016) and diagnostic category distribution (χ^2^ = 37.74, *p* < 0.001); a higher prevalence of males and mild Alzheimer Disease (AD) patients was found in Cluster 1 than Cluster 2. Cluster 1 had significantly lower cognitive reserve (U = 218.00, *p* = 0.003) and higher disease severity (U = 270.00, *p* = 0.006) than Cluster 2. Finally, the two clusters were also significantly different in terms of cognitive domains at T0: Cluster 2 outperformed Cluster 1 in global cognitive functioning (U = 0.00, *p* < 0.0001), long-term memory (U = 215.00, *p* < 0.001), logical-executive functions (U = 260.00, *p* = 0.01), and attention/processing speed (U = 244.00, *p* = 0.004). No other significant differences resulted between Cluster 1 and Cluster 2.

**Table 1:** Baseline patients’ characteristics as a function of cluster.

Characteristics		Cluster 1 (n = 27)	Cluster 2 (n = 30)	*p* value
Age	Mean ± std	74.22 ± 5.57	74.97 ± 5.58	0.321
Gender, female	n (%)	**12 (44)**	**23 (77)**	**0.016**
Years of education	Mean ± std	**8.56 ± 3.95**	**11.41 ± 4.24**	**0.006**
Diagnosis				** < 0.001**
PD-MCI	n (%)	**5 (19)**	**16 (53)**	
aMCI	n (%)	**2 (7)**	**11 (37)**	
mild AD	n (%)	**20 (74)**	**3 (10)**	
ADL	Mean ± std	5.88 ± 0.43	5.73 ± 0.78	0.232
IADL	Mean ± std	7.04 ± 1.15	7.30 ± 1.08	0.280
BDI	Mean ± std	6.58 ± 4.98	9.20 ± 6.07	0.110
CRIq	Mean ± std	**98.74 ± 17.55**	**112.50 ± 18.54**	**0.003**
CDRs	Mean ± std	**0.64 ± 0.27**	**0.50 ± 0.00**	**0.006**
Global cognitive functioning^+^	Mean ± std	**− 0.96 ± 0.50**	**0.76 ± 0.43**	** < 0.0001**
Attention/processing Speed^+^	Mean ± std	**0.14 ± 0.45**	**− 0.18 ± 0.45**	**0.004**

PD-MCI = Parkinson’s Disease Mild Cognitive Impairment; aMCI = amnesic Mild Cognitive Impairment; AD = Alzheimer’s Disease; ADL = Activities of Daily Living; IADL = Instrumental Activities of Daily Living; BDI = Beck Depression Inventory; CRIq = Cognitive Reserve Index Questionnaire; CDRs = Clinical Dementia Rating scale. ^+^denotes Z scores. Significant differences are bolded.

### Performance of the two clusters at the CoRe tasks

**Figure 1:**
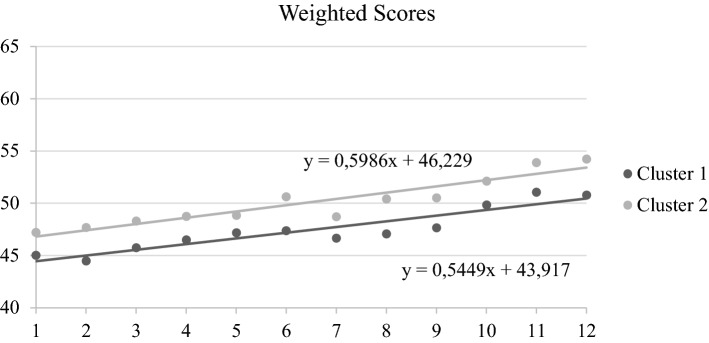
Slope and y intercept analyses referred to WSs in the CoRe tasks. *denotes significant differences between Cluster 1 and Cluster 2. X-axis refers to the number of the CoRe session (from session 1 to session 12); Y-axis refers to the reached WS.

We considered changes across sessions 1 to 12 in the *Weighted Score* (WS) of CoRe within each cluster. Both Cluster 1 (Z = − 4.37, *p* < 0.001) and Cluster 2 (Z = − 4.37, *p* < 0.001) reported a significant improvement across this period. We did not find any difference in the slope values (*p* = 0.69), and y intercepts for both clusters were positive, indicating an improvement across sessions. However, for Cluster 2 the y intercept resulted significantly higher compared to the y intercept of Cluster 1 (U = 157.00, *p* = 0.003) (see Fig. 1).

### Impact of CTT in global cognitive functioning and disease severity in the two clusters

**Figure 2:**
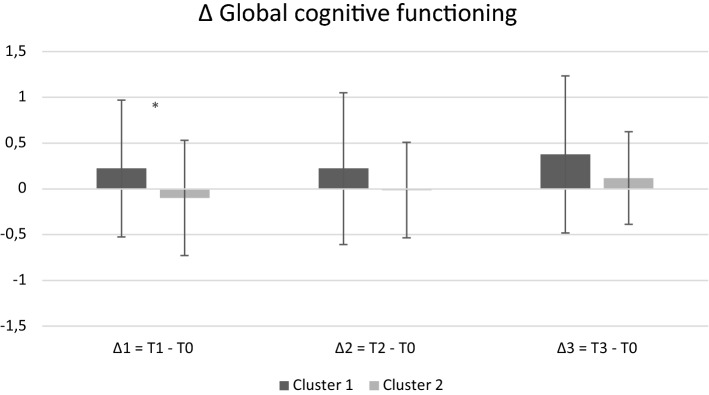
Delta scores for global cognitive functioning across testing sessions as a function of cluster. Positive values indicate improvement, while negative values indicate worsening across testing sessions. *denotes significant differences between Cluster 1 and Cluster 2. X-axis refers to each delta (∆1, ∆2, and ∆3); Y-axis refers to the value for each ∆ (z scores).

**Figure 3:**
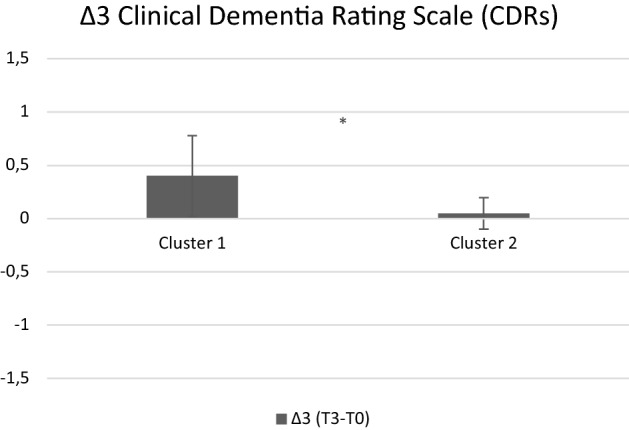
Delta scores for Clinical Dementia Rating scale across testing sessions as a function of cluster. Positive values indicate improvement, while negative values indicate worsening across testing sessions. *denotes significant differences between Cluster 1 and Cluster 2. X-axis refers to ∆3; Y-axis refers to the value for ∆3.

We evaluated differences between the two clusters in terms of delta scores (∆1 = T1-T0, ∆2 = T2-T0, ∆3 = T3-T0). As global cognitive functioning at ∆1, a significant difference was found between the two clusters (U = 271.00, *p* = 0.016): Cluster 1 gained more benefits than Cluster 2 after the intervention (see Fig. 2). A tendency to the same finding was found in ∆2 (U = 248.00, *p* = 0.07), but not in ∆3 (*p* = 0.15). As disease severity at ∆3, a significant difference was found in the Clinical Dementia Rating score (CDRs) (U = 36.00, *p* = 0.012), with Cluster 1 showing a more pronounced worsening in its disease severity than Cluster 2, which remained stable in its clinical condition (see Fig. 3).

Cores for global cognitive functioning and CDRs for Cluster 1 and Cluster 2 as a function of testing sessions can be found in Supplementary Fig. 1 and 2. Statistical comparisons of the cognitive domains, CDRs and CoRe WSs at each testing session are provided in Supplementary Table 1 and 2.

## Discussion

To the best of our knowledge, this is the first study exploring cognitive profiles in a sample of patients with early cognitive impairment as well as assessing the relationship between these profiles and the response to CCT. To this end, clusters were defined by considering both an index of global cognitive functioning comprising all cognitive abilities^19^ (i.e., MMSE), and a lower-order cognitive function involved in all other domains^20^ and sensitive to the effects of CCT^23,24^ (i.e., PS). The cluster analysis showed two relevant profiles displaying small within-cluster variation with the maximum between-cluster variation respectively: lower-functioning (Cluster 1) and higher-functioning (Cluster 2) cognitive profiles.

As demographic/individual characteristics, the lower-functioning profile group was composed mostly by patients with mild AD, low education, low cognitive reserve, and low cognitive performances at neuropsychological tests. By contrast, the higher-functioning profile group had a higher prevalence of MCI, individuals of female gender, high education, high cognitive reserve, and high cognitive performances. In addition, the two clusters at enrollment were also different in terms of CDRs, which is a clinician-rated staging method for cognitive dysfunction and functional ability^36^. Taken together, these findings confirm an association between higher/lower-functioning clusters and those demographic/individual characteristics involved in age-related cognitive processes^37^.

Both clusters improved in the CoRe WS (slope analysis). However, only Cluster 2 maintained higher scores throughout all sessions, as indicated by difference in y intercepts values of the two groups, which suggests that the cognitive profile of patients may affect the performance in the CCT tasks^38^. In any case, the adaptive regimen adopted by CoRe may have favored CCT improvements in both clusters by ensuring that the tasks were always challenging, cognitively demanding, and novel, accordingly to participants’ cognitive profiles.

Interestingly, even if Cluster 1 corresponded to a lower-functioning profile, it gained more post-intervention benefits in terms of global cognitive functioning (with a trend to stability at 6 months). The predictive power of individual differences regarding cognitive intervention responsiveness is often discussed within the compensation versus magnification framework^9^. The fact that the lower-functioning cognitive profile was associated to more pronounced changes in global cognitive functioning after CoRe intervention appears to be in line with the compensation account framework, and suggests that Cluster 1 subjects could have more “room for improvement” than individuals already performing at or near optimal levels, and therefore could show larger CCT benefits^9^. By contrast, Cluster 2 showed a stability in its clinical status one year from the end of CoRe intervention. Such a result seems to be in line with the magnification account: higher-functioning profile individuals having more resources to acquire, implement, and sharpen cognitive abilities would show longer lasting benefits, in particular for what concerns the severity of their disease. In other words, it seems that our results support both the compensation and magnification hypotheses. From one side, lower-functioning profile individuals gained a selective improvement in terms of “pure” cognitive functioning; from the other side, higher-functioning profile individuals, probably thanks to more favorable demographic/individual characteristics, obtained wider gains. Hence, our findings are in line with those^16^ suggesting that a high CR allows to cope better with neuropathology and to delay clinical manifestations of the neurodegenerative disease. The CDRs is indeed a rating device assessing dementia severity by staging, and includes cognitive, functional, and social domains in the overall staging^36^. These considerations imply that the validity of the magnification versus compensation account could at least partly vary according to the context (“pure” cognitive domain versus severity of the clinical status) in which it is applied^12^. In any case, it should be noted that Cluster 2 was composed by a higher prevalence of participants with MCI than Cluster 1. It is well known that cognitive intervention in MCI may represent a therapeutic option able to prevent or delay cognitive or functional decline^39^, whereas findings in relation to AD are still mixed^40^. Another explanation could be the more pronounced natural progression of the dementia stage which is more evident among individuals with mild AD than MCI^41^. Given the limited sample size, these results are purely speculative and larger studies are needed in order to confirm them.

This study has some limitations, which should be considered. First, as already mentioned, the small sample size, which did not allow clustering patients using a broader number of variables. In this context, we selected those we considered, according to the literature, more representative and sensitive to CCT effects^23,24^. Future studies could explore deeper the role of other predictors as clustering variables. Second, cluster analysis was conducted on the experimental group only. Therefore, the lack of a control group impedes disentangling predictors of CCT responsiveness from those of retest and practice effects^42^. Third, we enrolled patients aged 60–85. It should be noted that, in some cases, dementia-related impairment could manifest prior to older adulthood and that such a situation may underline different pathological processes^43,44^. Hence, our findings could not be generalized to all conditions of cognitive impairment.

In conclusion, this study provides a contribution to the identification of cognitive profiles associated with responsiveness to CCT, a promising non-pharmacological intervention option against cognitive dysfunction in neurodegenerative diseases. According to our findings, CCT might be especially beneficial for patients with MCI of higher education, and higher cognitive reserve to prevent or delay cognitive or functional decline. However, the observation that the lower-functioning profile was associated with a greater positive CCT responsiveness in terms of immediate specific cognitive functioning suggests that in this subgroup of patients longer lasting or repeated cycles of CCT may be required to maintain benefits. Hence, our findings suggest that compensatory and magnification effects are not mutually exclusive in explaining CCT improvements; indeed, they may both contribute to characterize and explain the outcome of these interventions. Taken together, clinicians based on these results should be able to predict the outcome for CCT based on patient’s characteristics and then, according to this, decide complementary approaches and treatments to be adopted.

## Methods

### Participants and measures

A total sample of 57 subjects (mean age = 74.61 ± 5.64) took part in this study. Participants were recruited and enrolled (March 2017-December 2019) from the Dementia Research Center outpatient services and Neurorehabilitation Unit of IRCCS Mondino Foundation (Pavia, Italy). The protocol was approved by the local ethics committee (San Matteo Hospital, Pavia, Italy) and registered in https://clinicaltrials.gov (NCT04111640). Written informed consent was collected from all the participants.

The inclusion criteria for patients were:

- A diagnosis of mild AD, amnesic MCI (aMCI), or Parkinson’s Disease (PD) MCI according to widely accepted diagnostic criteria (National Institute on Aging-Alzheimer’s Association (NIA-AA) criteria^45^ for mild AD; NIA-AA criteria^46^ for aMCI, and Movement Disorder Society (MDS) criteria^47^ for PD-MCI);
- Age between 60 and 85 years;
- Educational level ≥ 5 years;
- CDR^35^ score = 0.5–1, corresponding to patients with mild dementia or MCI.

The exclusion criteria were:

- MMSE score < 20
- Presence of cognitive impairment secondary to an acute or general medical disorders (e.g. brain trauma or tumor);
- Presence of severe neuropsychiatric conditions (e.g. mood and behavioral disorders);
- Presence of severe sensory (e.g. deafness or blindness) or motor impairments preventing trunk control and/or sitting position.

### Study design, procedures, and measures

At T0, the presence of cognitive impairment was formulated on the basis of a comprehensive neuropsychological evaluation according to literature^45–47^ and by using standardized tests adjusted to the Italian population. Particularly, we used the following tests and questionnaires:

- Global cognitive functioning: MMSE^48^;
- Working memory: Verbal Span, Digit Span, Corsi’s block-tapping test span^49^;
- Long-term memory: Logical Memory Test immediate-delayed recall^49,50^, Rey’s 15 words test immediate-delayed recall^51^, Rey Complex Figure delayed recall^52^;
- Logical-executive functions: Raven’s Matrices 1947^51^, Frontal Assessment Battery^53^, semantic^50^ and phonological fluencies (FAS)^51^, Rey Complex Figure copy^52^;
- Attention/processing speed: Attentive Matrices^49^, Trail Making Test^54^;
- Mood: Beck Depression Inventory (BDI)^55^;
- Functional status: Activities of Daily Living (ADL) and Instrumental Activities of Daily Living (IADL)^56^;
- Cognitive reserve: Cognitive Reserve Index questionnaire (CRIq)^57^;
- Disease severity: CDRs^35^.

After T0, patients received the CoRe intervention for 3 weeks. Follow-up evaluations were scheduled at T1 (global cognitive functioning), T2 (global cognitive functioning), and T3 (global cognitive functioning and CDRs).

### Interventions

CoRe is a software tool developed within a research project. It consists in 11 tasks targeting logical-executive functions, processing speed, working memory, and episodic memory (see Supplementary Table 3). The intervention comprised 4 face-to-face individual sessions per 3 weeks, each lasting 45 min. There is not a definitive consensus about the optimal number of session per week given the heterogeneity of the existing literature in the field of pathological aging^58^; hence we decided to reduce CCT total duration by increasing the number of session per week in order to optimize treatment adherence. During each session, participants were seated in front of a touch-screen computer and they were asked to perform the tasks, in randomized and counterbalanced order. The sessions took place in Neuropsychology Lab in the presence of a neuropsychologist. CoRe intervention is “adaptive”, which means that during the dynamic generation of the exercises, the individual patient’s performance data (accuracy and number of clues required) are analyzed in order to set the appropriate difficulty level. For each exercise and each level, thresholds are defined to allow difficulty levels to be progressively increased. Moreover, CoRe provides an overall performance indicator, so-called WS^27^, which is calculated taking into account different parameters: type of exercise, difficulty level, accuracy, response time. The WS serves to summarize the patient’s performance in the different exercises in a single value.

### Statistical analysis

The data reported in the present study pertain to the secondary analysis from a Randomized Controlled Trial (RCT) , whose primary outcome was the evaluation of cognitive changes following CoRe in early stage of cognitive impairment^28,29^. In the context of the present study, as primary-outcome measure we considered patients’ clustering. As secondary-outcome measures, we evaluated (1) demographic and individual characteristics of the two clusters; (2) performance of the two clusters at the CoRe tasks; and (3) impact of CTT in global cognitive functioning and disease severity in the two clusters. Sample size was not calculated given that there is no rule of thumb for minimum sample size for Cluster Analysis^59^.

Clustering is a powerful unsupervised machine learning technique often used in the medical field for discovering hidden patterns across patients’ characteristics that can be difficult to find for medical experts due to the number of variables to be considered simultaneously. We used the k-means clustering algorithm^60^ that considers *n* observations and divides them into *k* different sets such that the sum of distances between the observations and their respective cluster centroid is minimized. In our case, patients were subdivided by the k-means clustering algorithm in two groups according to their baseline standardized values of MMSE and PS. The choice of the number of clusters *k* can be influenced by prior knowledge of the data or driven by quantitative clustering quality measures such as the mean *silhouette* score. The mean silhouette score is a measure that considers both cohesion and separation of clusters and ranges between − 1 and 1. A value of 1 indicates that the clusters are cohesive and perfectly separated, a negative value indicates that the samples may be in the wrong cluster and a value close to 0 indicates overlapping clusters.

For the descriptive analysis of the clusters reported in Table 1 mean ($\overline{x }$) and SD were provided for all the continuous variables, while the number of participants in each category (n) and the percentage of patients over the total were considered for categorical variables. Considering the non-normal distribution of the collected measures, we used non-parametric tests for what concerned secondary-outcome measure analysis. We used the Mann–Whitney U test and Wilcoxon signed-rank test (continuous features) and Fisher’s exact test (categorical features) with a significance level of 0.05. We also fitted a linear function to the WS (dependent variable) obtained by patients at all 12 sessions (independent variable considered as equally spaced over time) to analyze differences in the increase rate (slope) and in the performance rate (y intercept) values between groups.

### Ethical approval

The study was performed in accordance with the guidelines of the Declaration of Helsinki. The study was approved by local ethics committee (San Matteo Hospital, Pavia, Italy).

## Supplementary Information

Supplementary Information.

## Data Availability

The datasets presented in this study can be found in online repositories. The names of the repository/repositories and accession number(s) can be found below: [10.5281zenodo.6476429].
