# Supplementary material for: Outcomes of a computer-based cognitive training (CoRe) in early phases of cognitive decline: a data-driven cluster analysis
Source: Sci Rep. 2023 Feb 7;13:2175. doi: 10.1038/s41598-022-26924-2 (PMC9905613; doi:10.1038/s41598-022-26924-2)
Supplement: Supplementary file 1 — Supplementary Information. [file 41598_2022_26924_MOESM1_ESM.pdf]

Outcomes of a computer-based cognitive training (CoRe) in early phases of cognitive decline: A data-driven cluster analysis

Sara Bernini[1], Alessia Gerbasi[2*], Silvia Panzarasa[2], Silvana Quaglini[2], Matteo Cotta Ramusino[1], Alfredo Costa[3,1], Micol Avenali[3,1], Cristina Tassorelli[3,1], Tomaso Vecchi[3,1], Sara Bottiroli[4,1]

[1]IRCCS Mondino Foundation, Pavia, 27100, Italy

[2]Dept. of Electrical, Computer and Biomedical Engineering, University of Pavia, Pavia, 27100, Italy

[3]Dept. of Brain and Behavioral Sciences, University of Pavia, Pavia, 27100, Italy

[4]Faculty of Law, Giustino Fortunato University, Benevento, 82100, Italy

[*] alessia.gerbasi01@universitadipavia.it

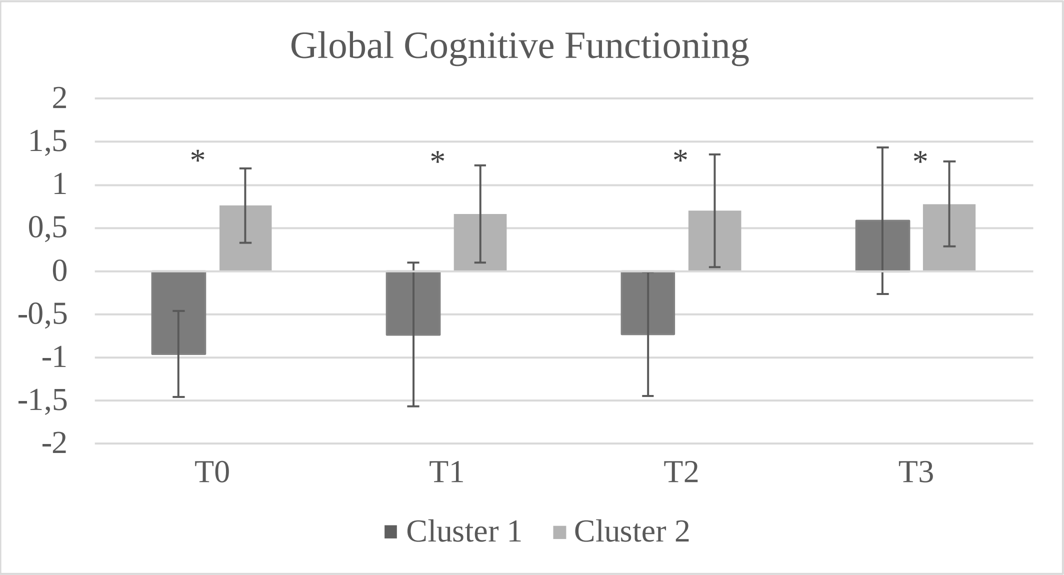

Supplementary Figure 1. Global cognitive functioning as a function of cluster and testing session.

*denotes significant differences between Cluster 1 and Cluster 2. X-axis refers to the timing of the testing session (T0, T1, T2, and T3); Y-axis refers to the global cognitive functioning performance in terms of z-scores.

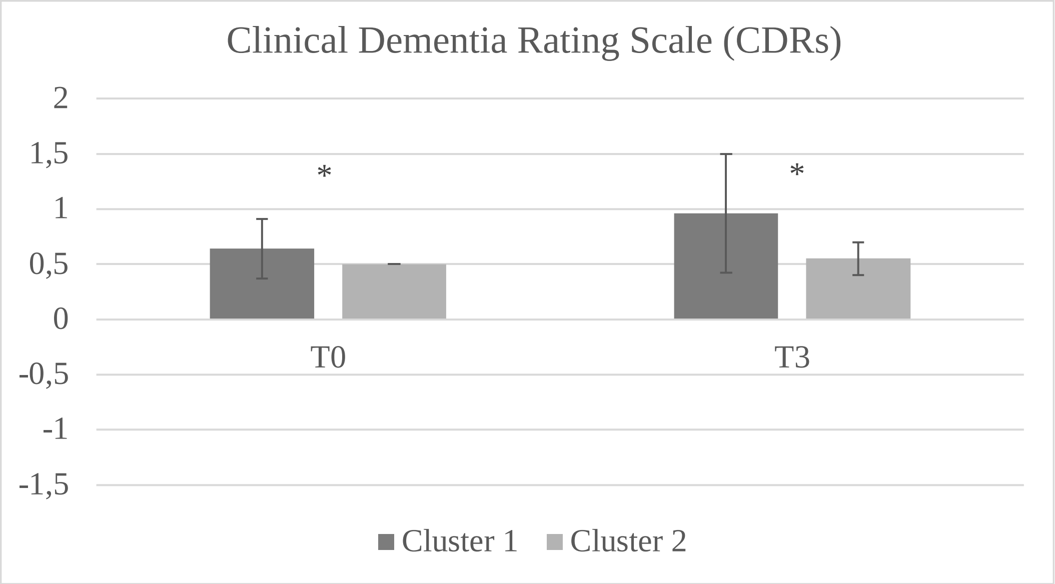

Supplementary Figure 2. Clinical Dementia Rating scale (CDRs) as a function of cluster and testing session.

*denotes significant differences between Cluster 1 and Cluster 2. X-axis refers to the timing of the testing session (T0 and T3); Y-axis refers to the CDR score.

Supplementary Table 1: Statistical comparisons of the cognitive domains (global cognitive functioning, working memory, long term memory, executive logic functions, attention/processing speed) and the CDRs at different testing sessions (T0, T1, T2, T3) as a function of cluster group.

	**Global cognitive functioning**	**Working memory**	**Long term memory**	**Executive logic functions**	**Attention/processing speed**	**CDRs**
**T0**						
Cluster1 (mean ± std)	**-0.961 ± 0.502**	-0.116 ± 0.711	**-0.351 ± 0.490**	**-0.235 ± 0.706**	**0.135 ± 0.452**	**0.394 ± 1.369**
Cluster 2 (mean ± std)	**0.763 ± 0.433**	0.133 ± 0.750	**0.276 ± 0.548**	**0.237 ± 0.691**	**-0.182 ± 0.450**	**-0.328 ± 0.000**
p-value	**<0.001**	0.102	**<0.001**	**0.010**	**0.004**	**0.003**
**T1**						
Cluster 1 (mean ± std)	**-0.737 ± 0.833**	**-0.235 ± 0.696**	**-0.461 ± 0.447**	**-0.241 ± 0.633**	**0.163 ± 0.431**	-
Cluster 2 (mean ± std)	**0.663 ± 0.561**	**0.211 ± 0.625**	**0.415 ± 0.748**	**0.217 ± 0.607**	**-0.146 ± 0.376**	-
p-value	**<0.001**	**0.008**	**<0.001**	**0.009**	**0.004**	-
**T2**						
Cluster 1 (mean ± std)	**-0.729 ± 0.718**	**-0.242 ± 0.605**	**-0.439 ± 0.458**	**-0.267 ± 0.639**	**0.095 ± 0.385**	-
Cluster 2 (mean ± std)	**0.701 ± 0.653**	**0.242 ± 0.660**	**0.439 ± 0.585**	**0.267 ± 0.534**	**-0.124 ± 0.484**	-
p-value	**<0.001**	**0.008**	**<0.001**	**0.003**	**0.048**	-
**T3**						
Cluster 1 (mean ± std)	**-0.585 ± 0.849**	-0.090 ± 0.671	**-0.250 ± 0.545**	**-0.194 ± 0.691**	**0.149 ± 0.382**	**0.424 ± 1.008**
Cluster 2 (mean ± std)	**0.780 ± 0.491**	0.121 ± 0.723	**0.322 ± 0.800**	**0.259 ± 0.714**	**-0.242 ± 0.470**	**-0.636 ± 0.450**
p-value	**<0.001**	0.299	**0.028**	**0.032**	**<0.001**	**0.009**

Note. Statistically differences are bolded.

Supplementary Table 2: CoRe WSs at session 1 and session 12 as a function of cluster group.

	WS
**Session 1**	
Cluster1 (mean ± std)	**45.279 ± 3.100**
Cluster 2 (mean ± std)	**47.102 ± 3.845**
p-value	**0.004**
**Session 12**	
Cluster 1 (mean ± std)	**56.551 ± 3.630**
Cluster 2 (mean ± std)	**58.702 ± 3.544**
p-value	**0.041**

Note. Statistically differences are bolded.

Supplementary Table 3: CoRe tasks' description, reporting involved cognitive skills.

TASKS	DESCRIPTION	MAIN INVOLVED SKILLS
LEARNING OF COUPLES	Pairs of words are shown on the screen, the patient must rewrite the second word of the couple when it is shown in a different order	Long-term memory abilities; learning and re-enactment strategies; visual imagery
WORD CATEGORIZATION	Words belonging to different categories are presented on the screen, the patient must rewrite them in any order but respecting the corresponding category	Long-term memory abilities; learning and re-enactment strategies; visual imagery; categorical thinking
PUZZLE	The patient must recompose the tiles to form a figure, the whole figure in the simplest levels is shown at the beginning of the exercise	Visuo-spatial long-term memory; visual imagery; mental representation and pianification
SPAN BACKWARDS	The patient must write the numbers in reverse order compared to how they were previously heard	Verbal working memory; processing-speed
MEMORY	Tiles that form pairs are shown on the screen, the tiles are turned and the patient has to choose two cards at a time to form all the pairs	Long-term memory abilities; visuo spatial abilities
VISUOSPATIAL MATRICES	The patient has to store and represent in the correct order on the grid the spatial instructions received (for example up, down, left, right, etc.)	Working memory; visuo-spatial abilities; processing-speed;
LOGICAL SEQUENCES	A sequence of images is shown, the patient must select, among several options, the one that completes the series	Non-verbal reasoning; mental problem solving; decision making
IMAGE AND SOUND	An image (small or big) is displayed and a sound (with low or high volume) is played; the patient must evaluate whether size and volume match	Inhibitory control; processing-speed; working memory;
UNSCRAMBLE THE SENTENCE	Scrambled words are displayed; the patient must select them in the right order to compose a sensible sentence	Mental and verbal planning; conceptual abstraction abilities
UNSCRAMBLE THE IMAGES	The patient must put the scrambled images in the right order to form a short story	Planning of activities: problem solving; temporal sequencing; visual attention
FIND THE ELEMENTS	A matrix of random elements (letters or numbers) is displayed, the patient must identify and select all the requested ones	Sustained and selective attention; visuo-spatial scanning; processing-speed
